# Cirsiliol induces autophagy and mitochondrial apoptosis through the AKT/FOXO1 axis and influences methotrexate resistance in osteosarcoma

**DOI:** 10.1186/s12967-023-04682-7

**Published:** 2023-12-12

**Authors:** Mengliang Luo, Zexin Su, Haotian Gao, Jianye Tan, Rongdong Liao, Jiancheng Yang, Lijun Lin

**Affiliations:** 1 Department of Joint and Orthopedics, Zhujiang Hospital, Southern Medical University, Guangzhou, 510282 China; 2 Department of Orthopaedics, The Second Affiliated Hospital of Nanchang University, Nanchang, 330006 China

**Keywords:** Osteosarcoma, Cirsiliol, Mitochondrial apoptosis, Autophagy, Chemoresistance

## Abstract

**Background:**

Osteosarcoma (OS) is the most common primary malignant bone tumor in children and adolescents, with poor outcomes for patients with metastatic disease or chemotherapy resistance. Cirsiliol is a recently found flavonoid with anti-tumor effects in various tumors. However, the effects of cirsiliol in the regulation of aggressive behaviors of OS remain unknown.

**Methods:**

The effect of cirsiliol on the proliferation of OS cells was detected using a cell counting kit-8 (CCK-8) assay and 5-ethynyl-2′-deoxyuridine (EdU) staining, while cell apoptosis was detected using flow cytometry. Immunofluorescence was applied to visualize the expression level of the mitochondria, lysosomes and microtubule-associated protein light chain 3 (LC3). A computational molecular docking technique was used to predict the interaction between cirsiliol and the AKT protein. The impact of cirsiliol on resistance was investigated by comparing it between a methotrexate (MTX)-sensitive OS cell line, U2OS, and a MTX-resistant OS cell line, U2OS/MTX. Finally, in situ xenogeneic tumor models were used to validate the anti-tumor effect of cirsiliol in OS.

**Results:**

Cirsiliol inhibited cell proliferation and induced apoptosis in both U2OS and U2OS/MTX300 OS cells. In addition, treatment with cirsiliol resulted in G2 phase arrest in U2OS/MTX300 and U2OS cells. Cell fluorescence probe staining results showed impaired mitochondria and increased autophagy in OS cells after treatment with cirsiliol. Mechanistically, it was found that cirsiliol targeted AKT by reducing the phosphorylation of AKT, which further activated the transcriptional activity of forkhead Box O transcription factor 1 (FOXO1), ultimately affecting the function of OS cells. Moreover, in situ tumorigenesis experiments showed that cirsiliol inhibited the tumorigenesis and progression of OS in vivo.

**Conclusions:**

Cirsiliol inhibits OS cell growth and induces cell apoptosis by reducing AKT phosphorylation and further promotes FOXO1 expression. These phenomena indicate that cirsiliol is a promising treatment option for OS.

**Supplementary Information:**

The online version contains supplementary material available at 10.1186/s12967-023-04682-7.

## Background

Osteosarcoma (OS) is the most widespread primary malignant tumor in orthopedics [1, 2]. The comprehensive treatment of surgery combined with chemotherapy has greatly improved the survival rate of patients with OS [3]. However, patients have a poor survival prognosis in the event of chemotherapy resistance and distant metastases [4, 5]. Therefore, identifying new drugs to treat OS is crucial.

Plant natural products are one of the solutions to the problems in modern medicine [6, 7]. Research has shown that various plant natural products are effective in treating tumors or delaying tumor progression [8, 9]. Cirsiliol is a flavonoid that is widely found in plants, such as *Leonotis nepetifolia* and *Artemisia*. It is reported that cirsiliol exerts multiple pharmacological effects, including anti-cancer, anti-inflammatory and anti-oxidative stress [10–13]. Although cirsiliol has been proven to be effective in treating esophageal squamous cell carcinoma and colon cancer [14, 15], the effect and underlying molecular mechanisms of cirsiliol in OS remain elusive.

Mitochondria are important organelles involved in energy generation, cell metabolism, and redox homeostasis, and constantly change their structure and morphology through a protein-mediated mechanism that controls fission and fusion processes [16, 17]. Under excessive mitochondrial fission, cells transfer the B-cell leukaemia/lymphoma 2 (BCL2) family member protein B-cell leukaemia/lymphoma 2-associated X (BAX) from the cytoplasm to the mitochondria, leading to an increase in the production of cytochrome *C* (Cyto-*C*). In addition, the damaged mitochondria are encapsulated by autophagosomes and degraded through the lysosomal pathway [18]. Several studies have shown that mitochondrial imbalance and altered autophagy levels affect tumor progression [14, 19, 20]. However, the precise underlying mechanisms in OS are yet to be elucidated. Therefore, the present study aimed to investigate whether cirsiliol can suppress OS progression and its possible underlying mechanisms.

## Materials and methods

### Cell culture

The human OS cell line U2OS was obtained from the American Type Culture Collection (ATCC, Manassas, VA, USA). The cell line was cultured in Dulbecco’s Modified Eagle Medium (DMEM) (Gibco, USA), containing 10% fetal bovine serum (Excell, Uruguay), and 1% penicillin G and streptomycin (Gibco, USA). MTX-resistant OS cell line U2OS/MTX was provided by Dr. M. Serra (Istituto Ortopedico Rizzoli, Bologna, Italy) and cultured in a medium containing 300 ng/mL MTX. All cells were incubated at 37 ℃ in a 5% CO_2_ atmosphere.

### Materials

Cirsiliol was purchased from Carbosynth (Oxford, UK) and prepared as a 30.3 mM stock solution with dimethyl sulfoxide solution (DMSO). SC79 (AKT activator) was acquired from MedChemExpress (New Jersey, USA) and dissolved in DMSO. Earle’s balanced salt solution (EBSS) was supplied by Sigma-Aldrich (USA) and dissolved in diethylpyrocarbonate (DEPC) water. In all cell experiments, the final concentration of DMSO did not exceed 0.5% in the medium. All drugs were stored at – 80 ℃ for a long time.

### siRNA transfection

U2OS and U2OS/MTX cells were plated at a density of 2 × 10^5^ cells per well in six-well plates. FOXO1 siRNA was obtained from Ige Biotechnology (Guangzhou, China), with the siFOXO1 sequence being 5′-UUAUCUCAGACAGACUGGGTT-3′. We transfected U2OS and U2OS/MTX cells with FOXO1 siRNA or control siRNA and incubated them at room temperature for 20 min. Subsequently, the mixture was introduced into the culture medium for use in subsequent experiments.

### Cell proliferation assay

U2OS and U2OS/MTX were inoculated at 1–2 × 10^3^ cells/well in a 96-well plate. Cirsiliol (at a concentration of 0, 5, 10, and 20 µM) was added to the wells after cell apposition. Cells were then assayed for proliferation using a cell counting kit-8 (CCK-8, Gibco, USA) assay. Approximately 100 µl of the reaction solution was added to each well. Next, cells were incubated at 37 ℃ in a 5% CO_2_ incubator for 2 h, and the absorbance was measured at 450 nm.

The viability of U2OS and U2OS/MTX cells was assessed by measuring EdU incorporation. EdU imaging Kit was purchased from ApexBio Technology (Houston, USA). Cell viability was assessed using fluorescence microscopy, where EdU-positive cells were marked in red, and nuclei were counterstained in blue.

### Cell cycle analysis

After 48 h of cirsiliol treatment, cells were routinely digested, washed 2–3 times with phosphate-buffered saline (PBS), and then fixed in pre-cooled 75% ethanol at 4 ℃ overnight. After propidium iodide (PI)/RNase staining (Dojindo, Japan), the cell cycle distribution was analyzed using CytoFLEX.

### Detection of apoptotic cells

To assess the extent of cirsiliol-induced apoptosis, the Annexin-V-FITC Apoptosis Detection Kit (Dojindo, Japan) was used to detect cell apoptosis ability. Cells were digested by EDTA-free trypsin and then incubated with FITC-labelled Annexin V and PI for 20 min. The result was analyzed using CytoFLEX.

### Western blot

Proteins were extracted from cells using the radioimmunoprecipitation assay (RIPA) buffer (Fudebio, China), which contained protein phosphatase inhibitors. Concentrations of protein were determined using a bicinchoninic acid (BCA) kit (Abbine, China). Equal amounts of protein were added to the loading buffer and boiled for 15 min. Proteins were then separated by sodium dodecyl sulfate–polyacrylamide gel electrophoresis (SDS-PAGE) and then transferred to polyvinylidene difluoride (PVDF) membranes in constant flow mode. The membranes were blocked in 5% skimmed milk powder at room temperature for 1 h and then incubated with specific antibodies at 4 ℃ overnight. The next day, membranes were washed in tris-buffered saline tween-20 (TBST) and incubated with secondary antibodies (Proteintech, China) at room temperature for 1 h. Finally, the protein signal intensity was detected with enhanced chemiluminescence (ECL) reagents (Millipore, USA). In this study, the primary antibodies used included anti-ACTB, anti-cleaved CASP3, anti-cleaved CASP9, anti-LC3B, anti-BECN1, anti-MFN2, anti-Cyto-C, anti-AKT, anti-p-AKT(Ser473) (Proteintech, China), anti-BAX, anti-BCL2(Abcam, USA), anti-FOXO1, anti-p-FOXO1(S256), anti-S6, anti-p-S6(236) (Cell signaling Technology, USA), anti-GSK3β(Affinity Biosciences, USA).

### Analysis of the mitochondrial transmembrane potential

Changes in the mitochondrial transmembrane potential after 48 h of cirsiliol treatment were assessed using the mitochondrial membrane Potential Assay Kit JC-1 (Beyotime, China). Briefly, cells were washed once with PBS, and 1 ml of JC-1 working solution was added to a six-well plate and incubated for 20 min. The fluorescent signal was then observed under AX NIS-Elements 5.4 (Nikon, Japan) Mitochondrial monomers and multimers were detected using CytoFLEX.

### Analysis of the mitochondrial morphology

Cells were stained for mitochondrial morphology using Mito-Tracker Red CMXRos (MTRC, Beyotime, China) and incubated with 25 nM mito-tracker for 20 min. Cells were rinsed and imaged using a confocal microscope.

### Immunofluorescence (IF) staining

Cells were treated with cirsiliol, fixed in 4% paraformaldehyde for 30 min and permeabilized by adding 0.2% Triton X-100 for 20 min. After incubation with 1% bovine serum albumin for 1 h, cells were incubated overnight with the corresponding primary antibody, followed by 2–3 washes with PBS buffer and incubation with the corresponding fluorescent secondary antibody for 1 h. Cell slides were sealed with an anti-fluorescent quencher containing 4′,6-diamidino-2-phenylindole (DAPI) and imaged using a confocal microscope.

### Construction of *in-situ* models

Animal experiments were approved by the Ethics Committee of Zhujiang Hospital, Southern Medical University. About 3–4 week-old female BALB/c-nu nude mice were purchased from Guangdong Medical Laboratory Animal Center and housed in a standard animal laboratory. Approximately 2 × 10^6^ cells transfected with luciferase plasmids were xenografted into the right tibia of the mice. When the mean tumor volume reached approximately 100 mm^3^, mice were randomly divided into two groups, with the control group given DMSO and the treatment group administered cirsiliol at 25 mg/kg. Mice were dosed every two days for a fortnight, and their weights and tumor volumes were measured. Then, mice were euthanized, and their organs and tumors were taken out for hematoxylin and eosin (H&E) staining and immunohistochemistry.

### Immunohistochemistry-paraffin (IHCP) assay

After being obtained, the tumor tissues were fixed in 4% paraformaldehyde for 48 h, dehydrated, embedded, and sectioned. The sections underwent routine dewaxing, hydration, and antigen repair. Tissues were incubated with primary antibodies against cleaved CASP3, Ki-67, PCNA. All antibodies were acquired from Proteintech(China). After two washes in PBS, sections were incubated with a secondary antibody for 1 h. 3,3′-diaminobenzidine (DAB) chromogenic agents were used to detect tissue antigens. The results were analyzed by microscopic image acquisition.

### H&E staining

Paraffin sections were dewaxed and hydrated. Afterward, cell nuclei were stained with hematoxylin for 3 min. Tissues were fractionated for 10 s, rinsed in pure water, and then immersed in eosin for 2 min. After undergoing treatment with pure water, alcohol, and xylene in turn, images were captured in a microscope.

### Molecular docking

A two-dimensional (2D) structure of cirsiliol (the ligand) was downloaded from PubMed and converted into a 3D structure using Chem3D. The protein structure of AKT was obtained from the Protein Data Bank (PDB, ID: 1H10). In this structure, water molecules were removed, and polar hydrogen atoms were added. Subsequently, molecular docking was performed using Autodock and the docking results were finally visualised by PyMOL.

### Statistical analysis

All data analyses were performed using SPSS (version 22.0) (IBM, USA). All experiments were performed with at least three biological replicates. The mean and standard deviation (SD) were calculated for quantitative variables, since they followed a normal distribution. Statistical differences were assessed using unpaired Student's t-test or one-way ANOVA. Images were statistically analyzed with FIJI/ImageJ. Flow patterns were analyzed using FlowJo 10.8.1. A P < 0.05 was considered statistically significant.

## Results

### Cirsiliol inhibits the proliferation and cell cycle of OS cells

**Fig. 1:**
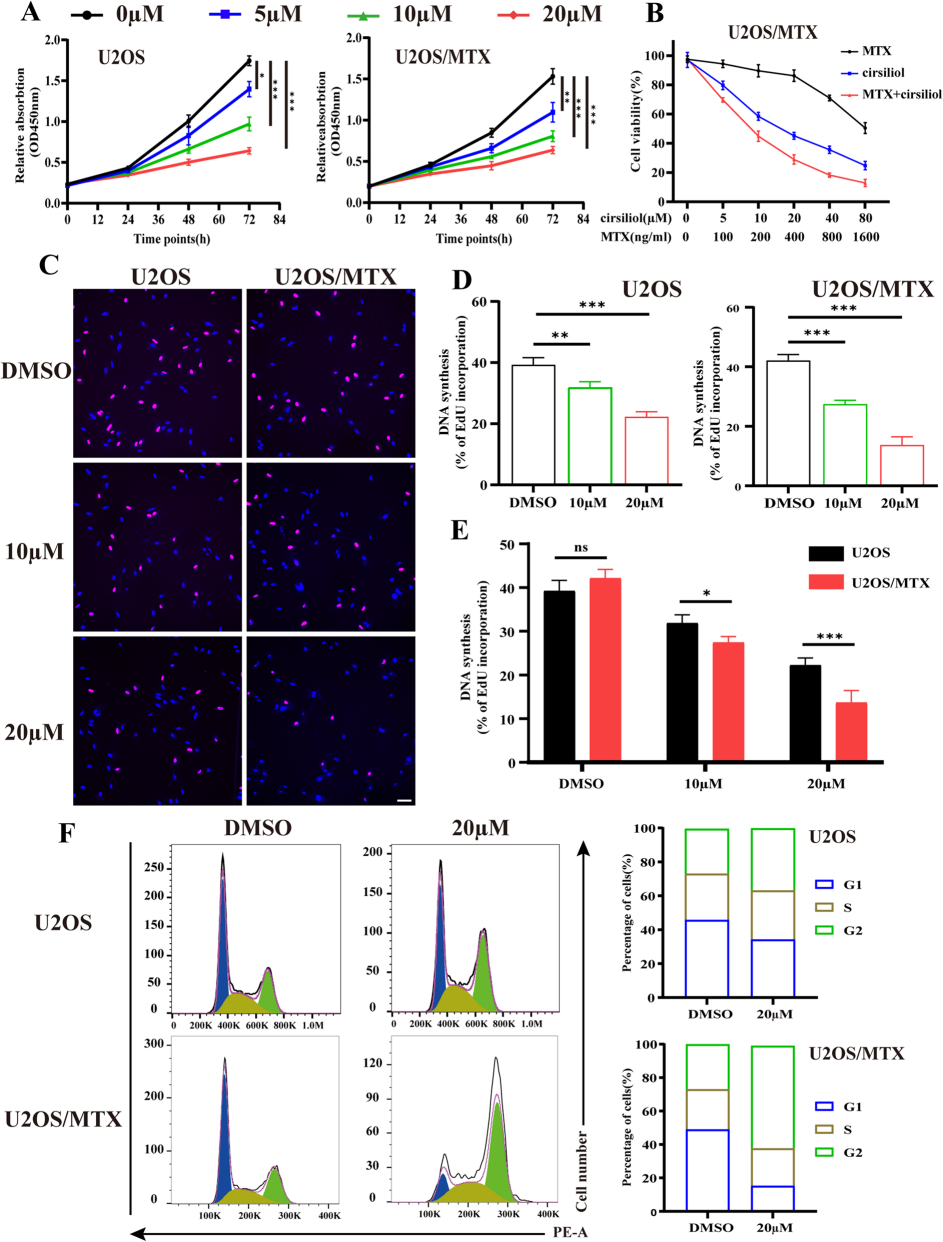
Cirsiliol inhibits cell proliferation and cell cycle in OS. **A** Cell proliferation was measured using the CCK-8 assay. U2OS and U2OS/MTX cells were treated with different concentrations of cirsiliol (0, 5, 10, 20 µM) for 72 h and absorbance values were measured at 450 nm for each group. **B** A CCK-8 assay was performed to assess the viability of U2OS/MTX cells treated with MTX and cirsiliol at each designated concentration for 48 h. **C**, **D**. U2OS and U2OS/MTX cells were treated with cirsiliol (0, 10, 20 µM) to measure the expression of proliferating DNA. Three independent experiments were performed. Scale bars: 100 µm. **E**. Under the same concentration of cirsiliol treatment, a statistical plot of the difference in cell proliferation between U2OS and U2OS/MTX cells was shown. **F** U2OS and U2OS/MTX cells were treated with DMSO (20 µM) for 48 h. The distribution of cell cycles was measured using flow cytometry. Data are expressed as mean ± SD. *p < 0.05; **p < 0.01; ***p < 0.001

OS cell lines U2OS and U2OS/MTX (continuously exposed to 300 ng/ml of MTX) were used to assess the effect of cirsiliol on OS cell growth. Cells were exposed to different concentrations of cirsiliol (0, 5, 10, and 20 µM for 48 h). The results of the CCK-8 assay showed that cirsiliol intervention significantly reduced cell proliferation (Fig. 1A). Meanwhile, the therapeutic effect of the combination of MTX and cirsiliol was enhanced compared to MTX or cirsiliol alone (Fig. 1B). Moreover, EdU staining results revealed that the amount of red fluorescently-labeled DNA also decreased (Fig. 1C, D). Together, these findings indicate that cirsiliol has a significant inhibitory effect on the proliferation of OS cells. Interestingly, U2OS/MTX was more sensitive to the drug than U2OS under the same concentration of cirsiliol treatment (Fig. 1E).

Furthermore, cell cycle checkpoints operate as DNA surveillance mechanisms that prevent the accumulation and propagation of genetic errors during cell division [21]. Thus, we assessed the effect of cirsiliol on the cell cycle. It was found that the number of cells in the G1 phase was significantly reduced in OS cell lines compared with the DMSO group, while the G2M phase exhibited the opposite result (Fig. 1F). This further demonstrates that cirsiliol can affect the proliferative capacity of OS cells by blocking the cell cycle.

### Cirsiliol induces apoptosis in OS cells

**Fig. 2:**
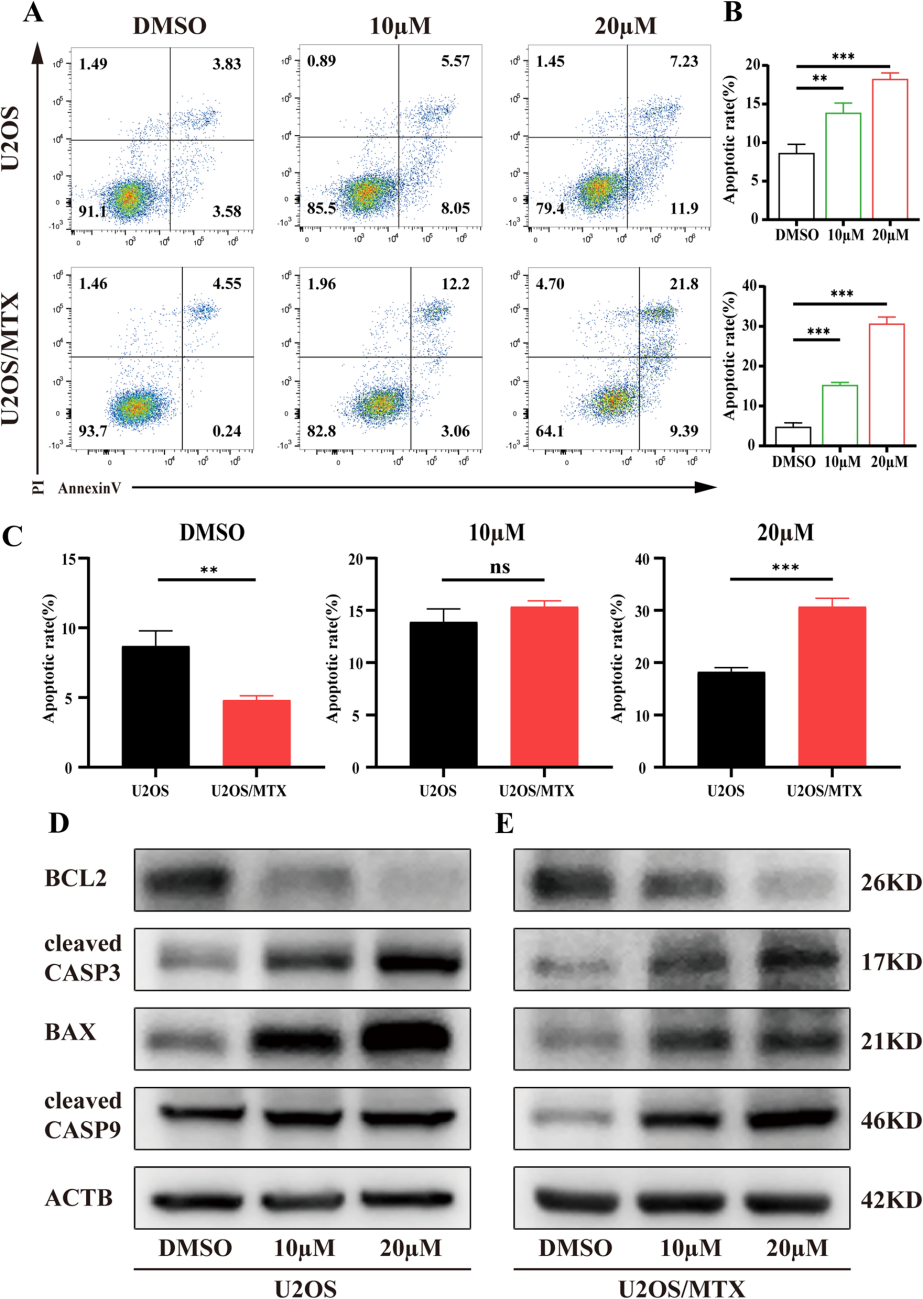
Cirsiliol induces apoptosis in OS cells. **A** Annexin-V-FITC staining was used to determine the proportion of apoptosis occurring in U2OS and U2OS/MTX cells treated with DMSO (10 and 20 µM). **B** Statistical plots of early and late apoptosis in U2OS versus U2OS/MTX at different concentrations of cirsiliol for three replicate experiments. **C** Under the same concentration of cirsiliol treatment, a statistical plot of the difference in cell apoptosis between U2OS and U2OS/MTX cells was shown. **D**, **E** Western blot analysis of lysates from cirsiliol-treated U2OS or U2OS/MTX with BCL2, cleaved CASP3, cleaved CASP9, BAX and ACTB antibodies. Data are expressed mean ± SD. *p < 0.05; **p < 0.01; ***p < 0.001

**Fig. 3:**
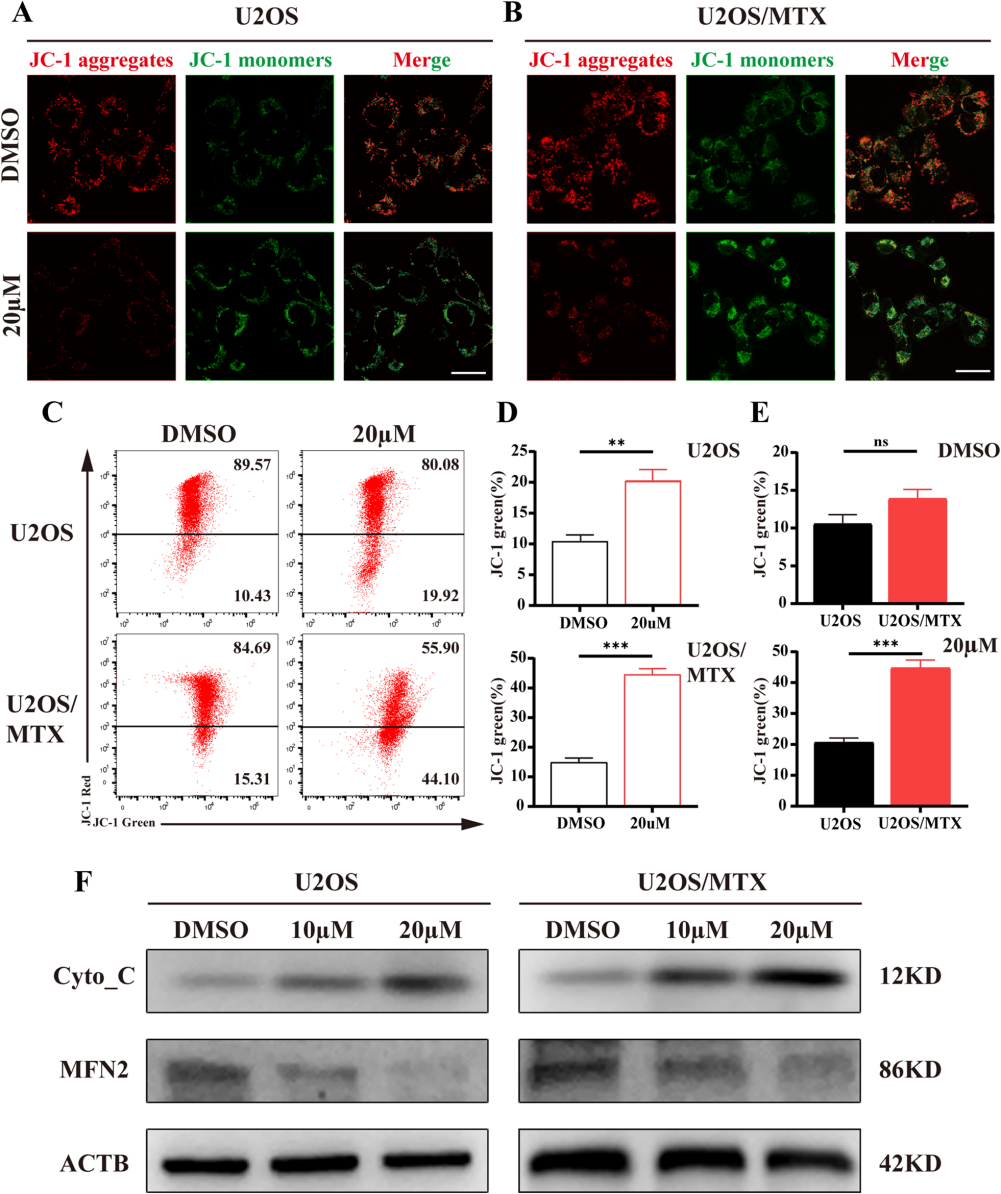
Cirsiliol affects the mitochondrial membrane potential of osteosarcoma cells. **A**, **B** U2OS and U2OS/MTX cells were treated with DMSO (20 µM), respectively, and incubated with JC-1 for 30 min. Representative confocal microscopy images. Scale bar: 100 µm. **C** U2OS and U2OS/MTX cells were treated with DMSO (20 µM), respectively, and incubated with JC-1 for 30 min. The distribution of JC-1 aggregates and monomers was examined by flow cytometry. **D** Flow cytometry analysis of JC-1 monomers and JC-1 aggregates at different concentrations. **E** Statistical plot of the difference in mitochondrial membrane potential between U2OS and U2OS/MTX cells under the same concentration of cirsiliol treatment. **F** Western blot analysis of lysates from cirsiliol-treated U2OS and U2OS/MTX cells against Cyto-C, MFN2 and ACTB antibodies. Data are expressed as mean ± SD. *p < 0.05; **p < 0.01; ***p < 0.001

Tumor cell growth and dysregulation of the cell cycle effectively promote apoptosis [22]. Therefore, Annexin-V-FITC staining and flow cytometry were used to evaluate the apoptotic effect of cirsiliol on U2OS and U2OS/MTX cells. The rate of cell apoptosis increased with increasing concentrations of cirsiliol and was statistically significantly different (Fig. 2A, B). Moreover, the rate of apoptosis in U2OS/MTX cells noticeably rose when treated with 20 µM of cirsiliol, corroborating that cirsiliol correlates with methotrexate resistance (Fig. 2C). It has been suggested that the possible pathways involved in apoptosis are the mitochondrial pathway, the death receptor pathway and the endoplasmic reticulum pathway. Hence, western blot analysis was performed to further determine the pathway through which cirsiliol affects apoptosis in OS cells. Results showed that the expression of all three important markers of the mitochondrial pathway, BAX, Cyto-C and cleaved CASP9 was dramatically increased (Figs. 2D, E, 3F), while that of BCL2—which inhibits the mitochondrial membrane permeabilization by pro-apoptotic proteins BAX and BCL2 antagonist/killer 1 (BAK)—was decreased (Fig. 2D, E). Collectively, these results suggest that cirsiliol affects cell apoptosis mainly through the mitochondrial pathway.

### Cirsiliol promotes mitochondrial fission and apoptosis

**Fig. 4:**
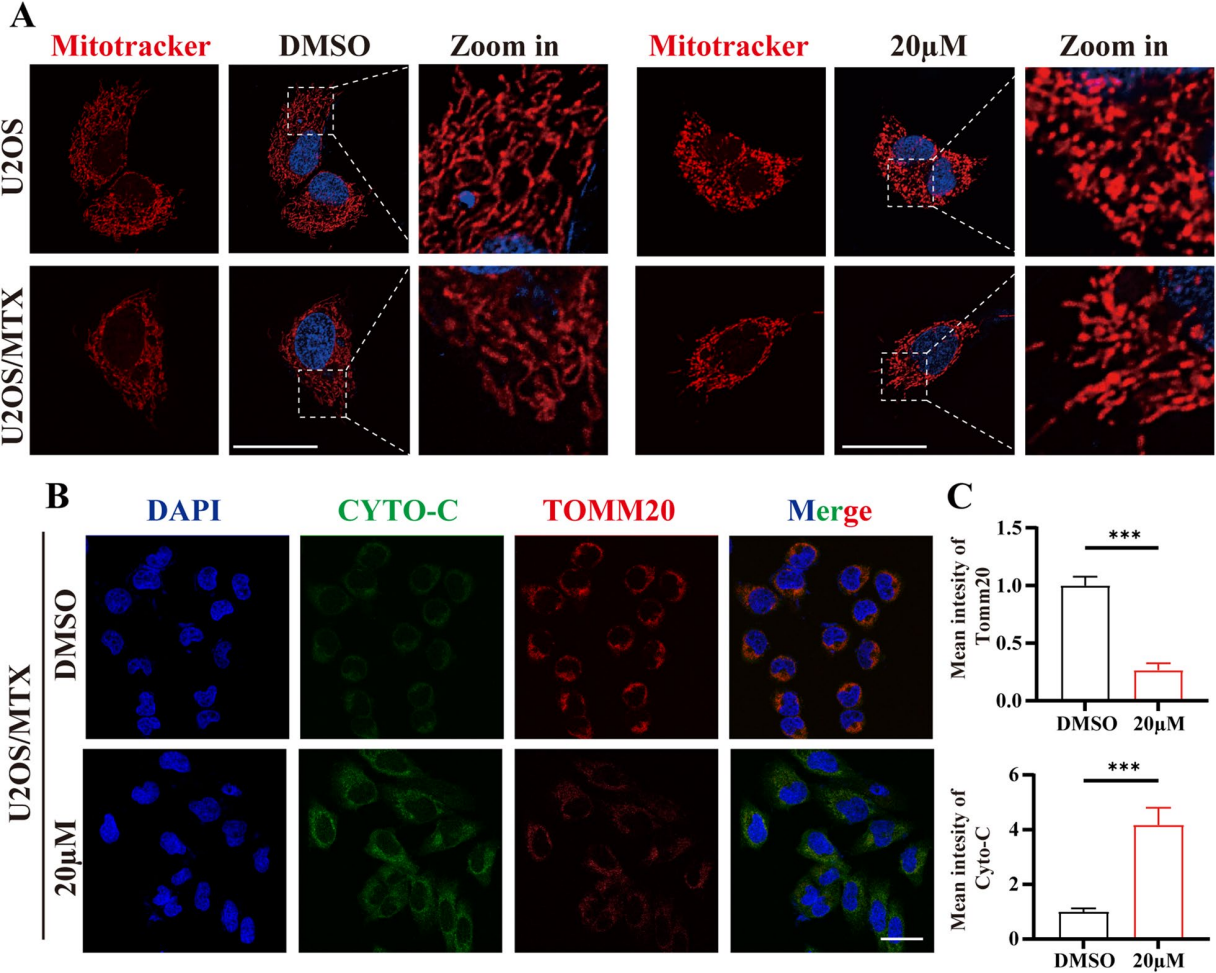
Cirsiliol promotes mitochondrial fission and apoptosis. **A** U2OS and U2OS/MTX cells were treated with DMSO (20 µM), and then stained with MTRC, respectively. **B**, **C** U2OS/MTX cells were treated with various concentrations of cirsiliol, stained with anti-Cyto-C and anti-TOMM20 antibodies, and imaged using co-focused microscope imaging. Scale bars:100 µm. Cyto-C and TOMM20 were quantified using FIJI/ImageJ. Data are expressed as mean ± SD. *p < 0.05; **p < 0.01; ***p < 0.001

Mitochondrial fusions and fissions are in relative equilibrium that can be altered by external stimuli or interference with fusion and fission indicators. Therefore, we first examined the mitochondrial membrane potential (ΔΨm) using fluorescence microscopy. The results revealed that the presence of cirsiliol significantly decreased ΔΨm (Fig. 3A, B). Similar results were obtained under flow cytometry (Fig. 3C, D). Meanwhile, changes in the expression of mitofusin 2 (MFN2), a mitochondrial fusion index, further affected the homeostasis of ΔΨm (Fig. 3F). The statistical results showed that U2OS/MTX was more sensitive to cirsiliol (Fig. 3E). Next, due to the imbalance in mitochondrial transmembrane potential, we used MTRC to label the mitochondria and noted fragmented mitochondria accumulated after cirsiliol treatment. Additionally, the mitochondrial length and number of branches reduced substantially compared with the DMSO group (Fig. 4A). These phenomena indicate that the structure and function of the mitochondria were damaged.

It has been reported that a shift in mitochondrial membrane permeability is closely related to apoptosis [23, 24]. Abnormal opening of the mitochondrial permeability transition pore (MPTP) leads to the rupture of the outer mitochondrial membrane and swelling of the matrix. Under such conditions, the apoptosis-inducing factor of the membrane gap and Cyto-C (a key substance for mitochondrial apoptosis) are released into the cytoplasm, which triggers the entire apoptotic program. The increased expression of Cyto-C in response to cirsiliol treatment was initially verified by western blot analysis (Fig. 3F). In addition, we co-stained Cyto-C with translocase of outer mitochondrial membrane 20 (TOMM20) (Fig. 4B, C) (Additional file 1: Fig. S1A, B) and found a consistent phenomenon. Overall, these results confirm that cirsiliol can impair mitochondrial function and its apoptosis occurrence.

### Cirsiliol-induced apoptosis is associated with autophagy in OS cells

**Fig. 5:**
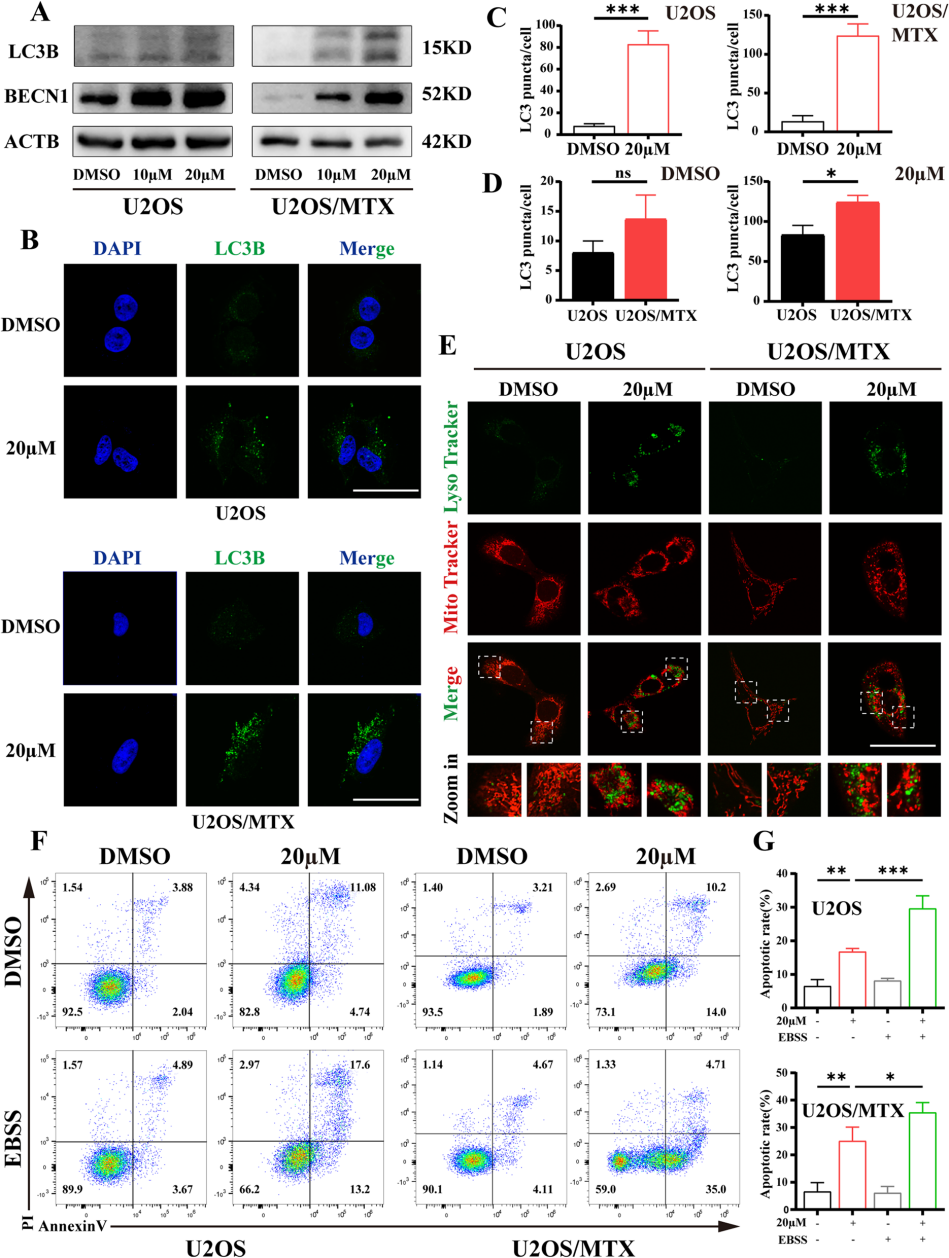
Cirsiliol-induced apoptosis is associated with autophagy in OS cells. **A** U2OS or U2OS/MTX cells were treated with DMSO (10 and 20 µM) at three concentrations and lysates were extracted, and western blot analysis was used to measure protein expression of LC3B, BECN1 and ACTB. **B** Immunofluorescence analysis of the number of LC3B spots formed by U2OS or U2OS/MTX cells after treatment with DMSO (20 µM). Scale bar: 50 µm. **C** Representative statistical analysis graph of **B** using FIJI/ImageJ. Five to 10 fields of view were taken, and three replicate experiments were performed. **D** Graphical representation of the variance in autophagy levels between U2OS and U2OS/MTX cells treated with an equivalent concentration of cirsiliol. **E** Lysosomes were labeled using LTG and then co-stain with MTRC to observe changes in the mitochondrial structure and the number of lysosomes in U2OS or U2OS/MTX cells. Scale bar: 50 µm. **F**, **G**. U2OS or U2OS/MTX cells were treated with permutations of cirsiliol and/or EBSS for 48 h. The proportion of apoptosis was detected by Annexin-V-FITC staining. Data are expressed as mean ± SD. *p < 0.05; **p < 0.01; ***p < 0.001

It has been documented that autophagy removes damaged organelles and degrades them via the lysosomes [25, 26]. At present, whether autophagy affects apoptosis in OS cells is yet to be understood. Therefore, we explored the molecular targets related to autophagy and detected the protein expression of LC3B and beclin1 (BECN1) using western blot analysis. The results showed that cirsiliol upregulated the level of autophagy in OS cells (Fig. 5A). Meanwhile, endogenous LC3B staining was applied to analyze autophagosome formation. The results showed a significant accumulation of positive spot-like structures of LC3B in the 20 µM cirsiliol group (Fig. 5B, C). In addition, the level of autophagy was more pronouncedly elevated in U2OS/MTX cells under cirsiliol treatment compared to U2OS cells (Fig. 5D). The accumulation of autophagosomes may impair lysosomal functions. Next, lysosomes were labeled using Lyso-Tracker Green (LTG) and then co-stained with MTRC (Fig. 5E). It was found that the LTG signal was significantly elevated in lysosomes when the mitochondria were in a poor state. Taken together, these data suggest that cirsiliol promotes the accumulation of autophagosomes in OS cells.

Previous reports have indicated that autophagy is generally a protective factor against tumor development [27]. EBSS alone or in combination with cirsiliol were used to elucidate the effect of autophagy on the apoptotic process in OS cells. It was found that treatment with EBSS alone increased the degree of apoptosis in OS cells compared with the cirsiliol-treated group (Fig. 5F, G). These findings reveal that cirsiliol has a detrimental effect on OS cell apoptosis and autophagy.

### Cirsiliol exerts its action through the AKT/FOXO1 axis

**Fig. 6:**
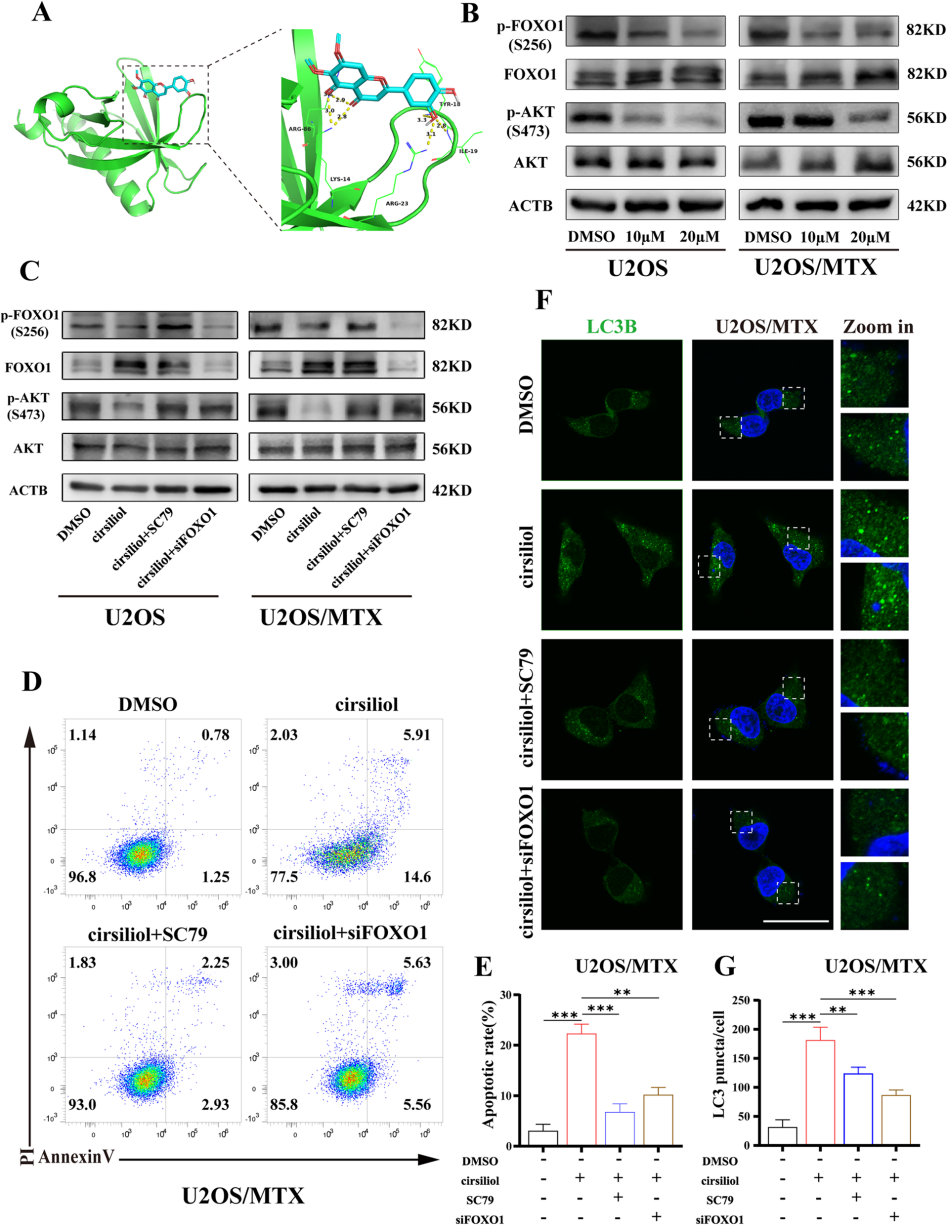
Cirsiliol exerts its action through the AKT/FOXO1 axis. **A** Molecular docking model of cirsiliol versus AKT. **B** Western blot assays were performed on lysates from U2OS and U2OS/MTX cells treated with cirsiliol. The antibodies used included AKT, p-AKT, FOXO1, p-FOXO1, and ACTB. **C** SC79 and siFOXO1 were added to detect the expression levels of AKT, p-AKT, FOXO1, p-FOXO1, and ACTB. **D** Incidence of cell apoptosis in groups DMSO, cirsiliol (20 µM), cirsiliol + SC79, and cirsiliol + siFOXO1. **E** Statistical graphs done for **D**. Three replicate experiments were performed. **F**, **G** Immunofluorescence staining was performed to verify the difference in LC3B expression among the DMSO, cirsiliol (20 µM), cirsiliol + SC79, and cirsiliol + siFOXO1 groups. Scale bars: 50 µm. The number of spots was counted on the fluorescent images using FIJI/Image J. Data are expressed as mean ± SD. *p < 0.05; **p < 0.01; ***p < 0.001

The phosphoinositide 3-kinase (PI3K)/AKT pathway is one of the major signaling pathways associated with tumor proliferation and migration [28]. Therefore, we performed molecular docking using AutoDock Tools. The results showed that cirsiliol had hydrogen bond interactions with amino acid residues ARG-86, TYR-18, LYS-14, ILE-19 and ARG-23 on the AKT domain (Fig. 6A). Molecular docking showed that cirsiliol and AKT had robust interactions and strong binding affinity (− 6.1 kcal/mol). To further determine whether there was involvement of the PI3K/AKT pathway, the expression of AKT and phosphorylated AKT was examined using western blot analysis (Fig. 6B). Phosphorylated AKT signals were significantly downregulated, indicating that cirsiliol acts as an inhibitor of the PI3K/AKT pathway.

However, we further explored the mechanism of autophagosome formation. Some canonical downstream proteins of PI3K/AKT pathway were validated, including mTOR, GSK3β, and FOXO1. The results indicated that the protein expression level of FOXO1 was significantly up-regulated in response to changes in the concentration of cirsiliol (Fig. 6B; Additional file 2: Fig. S2A). Moreover, previous studies demonstrated that FOXO1 promoted the expression of several autophagy-related genes, and post-translational modifications of FOXO1 are required for triggering the autophagic process [29]. And then, IF staining revealed that cirsiliol significantly increased the number of autophagosomes in both U2OS and U2OS/MTX cells (Fig. 6F, G; Additional file 3: Fig. S3B, D).

To further validate that cirsiliol affects osteosarcoma through the AKT/FOXO1 pathway. We added SC79, an activator of AKT. The results showed that osteosarcoma cell proliferation was upregulated (Additional file 2: Fig. S2B, C), the rate of apoptosis was decreased and the number of autophagosomes was significantly reduced (Fig. 6D, E; Additional file 3: S3A, C). Meanwhile, We got similar results when we knocked down FOXO1.

Taken together, these findings suggest that cirsiliol can promote apoptosis as well as the accumulation of autophagosomes by modulating the AKT/FOXO1 axis. Interestingly, various results confirmed that cirsiliol significantly inhibited cell proliferation, promoted cell apoptosis, and led to autophagosome accumulation in U2OS/MTX compared to U2OS cells. It is therefore reasonable to assume that cirsiliol is more effective in U2OS/MTX.

### Cirsiliol inhibits xenogeneic OS tumor growth in situ

**Fig. 7:**
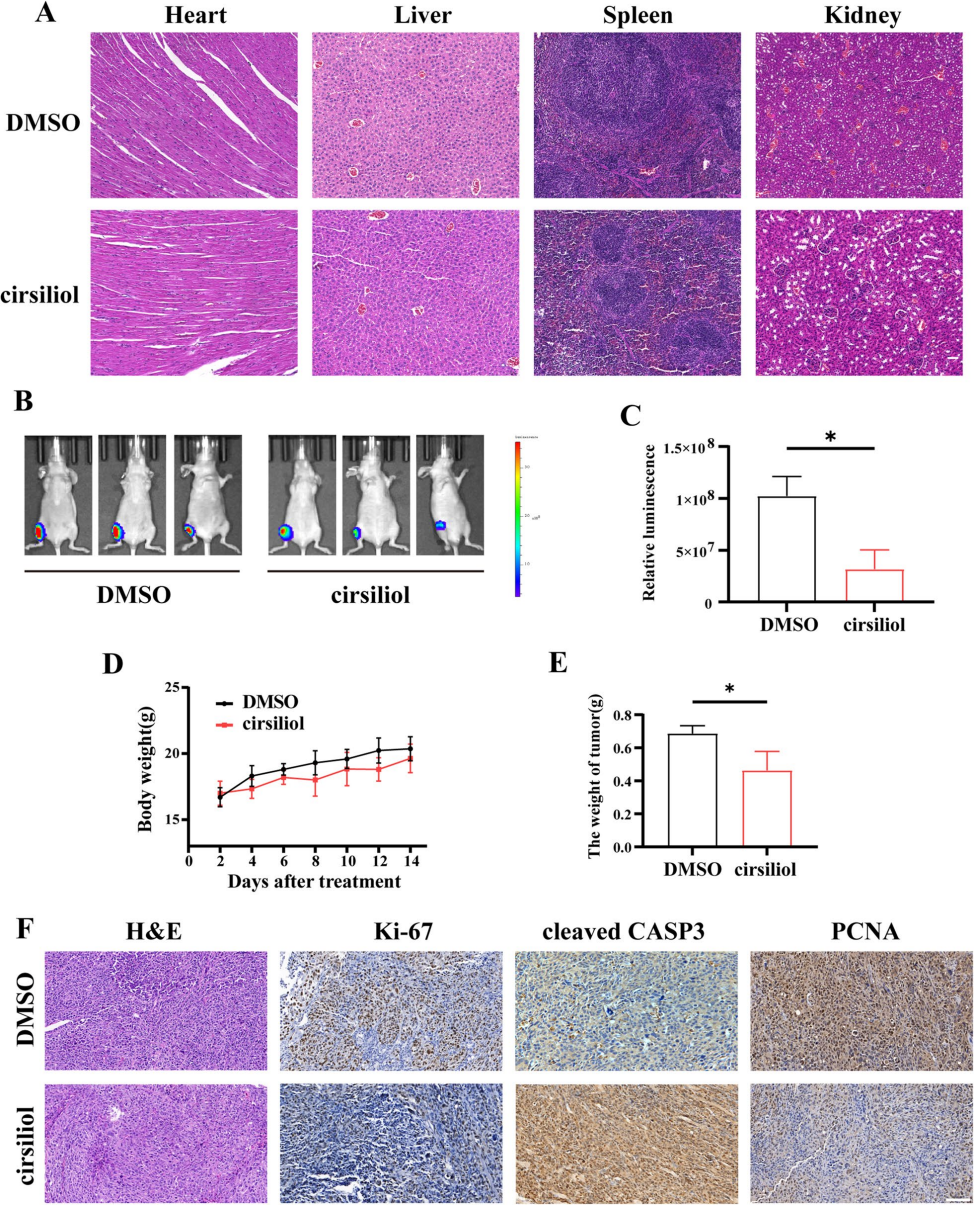
Cirsiliol inhibits xenogeneic OS tumor growth in situ. **A** H&E staining of major organs. Scale: 50 µm. **B**, **C** A small animal in vivo optical imaging system was utilized to detect fluorescence intensity values after 14 days of continuous treatment with cirsiliol. **D** Body weight curves were measured for mice in different treatment groups (n = 3 per group). **E** Statistical analysis of the final tumor volume and size in different treatment groups. **F** IHCP analysis of the expression of KI67, PCNA and cleaved CASP3 in tumor tissues and the results from H&E staining in treatment and control groups. Scale bar: 100 µm. Data are expressed as mean ± SD. *p < 0.05; **p < 0.01; ***p < 0.001

To determine the effect of cirsiliol on OS in vivo, we constructed a mouse in situ xenograft tumor model. After 2 weeks, tumor-bearing mice were randomized into two groups, with intraperitoneal administration of DMSO and 25 mg/kg of cirsiliol, respectively. In vivo experiments showed that cirsiliol mitigated the growth of OS in situ (Fig. 7B, C). In addition, no significant weight loss was observed in the experimental mice (Fig. 7D). At the end of the experiment, the tumors were taken out and weighed, and it was found that tumor weight differed significantly between the two groups (Fig. 7E). Moreover, to determine the potential toxicity of the drug to normal organs, vital organs of mice were collected and subjected to H&E staining. There was no major organ-related toxicity in the cirsiliol group (Fig. 7A). Subsequently, we conducted IHCP staining utilizing markers associated with proliferation, such as Ki-67 and proliferating cell nuclear antigen (PCNA), as well as the apoptosis-related indicator cleaved caspase3(cleaved CASP3). The results indicated that cirsiliol not only inhibited proliferation but also induced apoptosis in OS cells (Fig. 7F).

## Discussion

Over the past few years, some progress has been made in improving survival outcomes for patients with OS [30]. However, treatment of OS is still faced with several pressing issues, including the development of rapidly progressive disease, distant metastases and drug resistance. Recently researchers have been committed to find effective treatment methods for drug-resistant patients with osteosarcoma. Indeed, osteosarcoma cells are known to express a number of surface proteins that might be of therapeutic relevance, including B7-H3, GD2 and HER2, which can be targeted using antibody–drug conjugates or adoptive cell therapies [31–33]. In addition, The emergence of new targeted drugs targeting classic oncogenes has also provided new options for the treatment of osteosarcoma, such as KRAS. Studies have proved that KRAS could promote proliferation of osteosarcoma cells and induced cisplatin resistance. Furthermore, KRAS has also been proven to be associated with drug resistance in various tumors [34–37]. With all of the new biological discoveries, technologies, agents and approaches, the treatment of osteosarcoma patients seems to be approaching a breakthrough [38, 39].

**Fig. 8:**
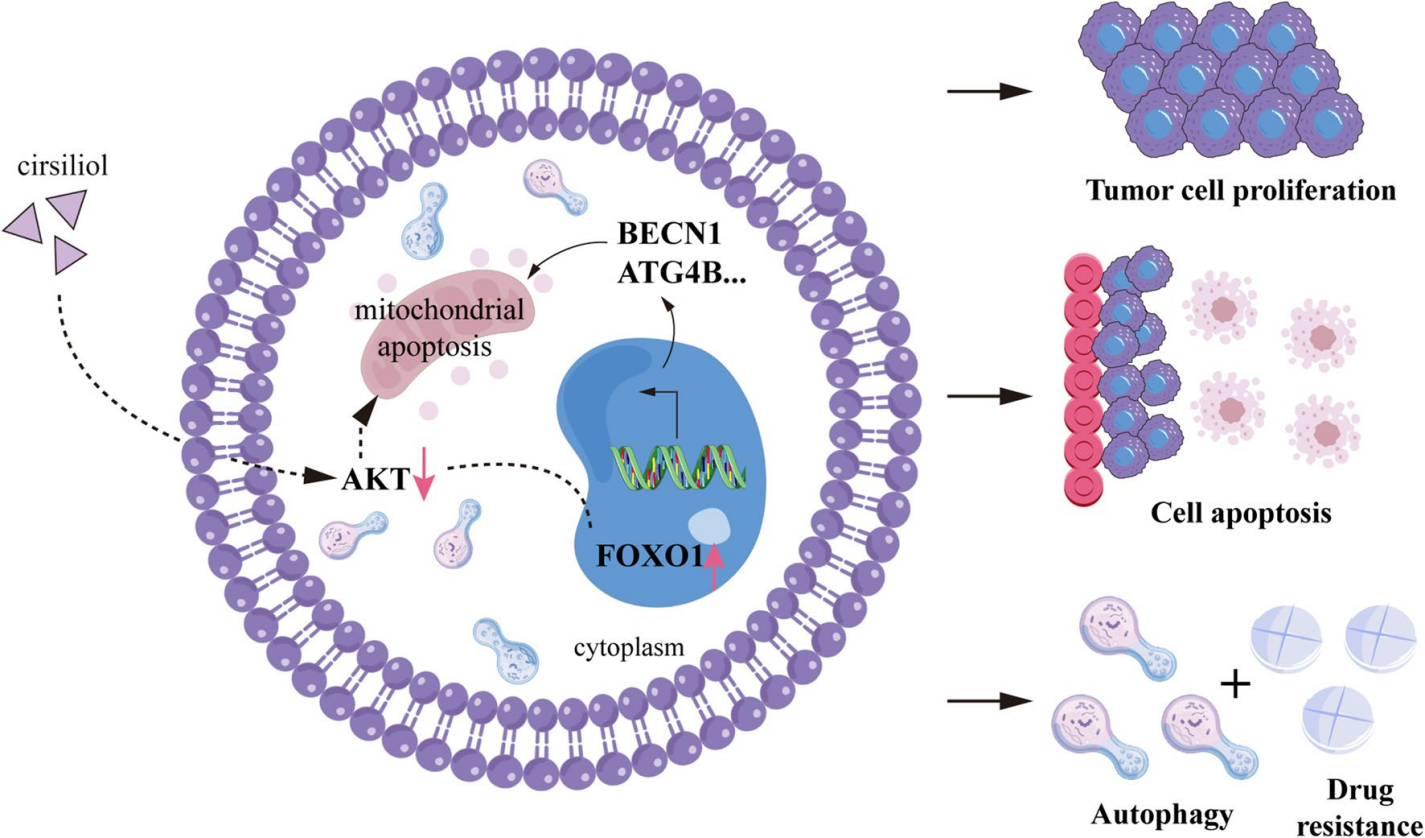
Schematic diagram illustrating cirsiliol-induced autophagy and mitochondrial apoptosis through the AKT/FOXO1 axis, and its impact on methotrexate resistance in osteosarcoma

Cirsiliol is a flavonoid that has been reported to have therapeutic effects on esophageal squamous cell carcinoma, melanoma, non-small cell lung cancer and breast cancer [13, 15, 40, 41]. It may affect mitochondrial function and ATP generation or target the appropriate protein to exert its inhibitory effects [14, 42]. However, the mechanism of action of cirsiliol in OS is unknown, warranting further investigation, particularly in resistant cell lines. The present study validated the antitumor properties of cirsiliol against OS in vivo and in vitro. We observed that cirsiliol led to a reduction in cell proliferation and an elevation in apoptosis and autophagy levels in U2OS/MTX compared with U2OS cells. Further, we found that cirsiliol acts on OS through the AKT/FOXO1 axis by affecting the phenotype associated with OS (Fig. 8, by Figdraw).

Our study found that cirsiliol regulates the apoptosis of OS cells in a concentration-dependent manner. To verify the pathway through which cirsiliol causes apoptosis, the mitochondrial pathway, also known as the endogenous apoptotic pathway, was first tested. Mitochondria are the main site of apoptosis regulation and are involved in most cell apoptotic processes [43]. We found that cirsiliol not only disrupted mitochondrial morphology but also affected its function and activity. Meanwhile, Cyto-C is released in large quantities from the mitochondrial matrix into the cytoplasm, activating the apoptosis promoter caspase-9 and eventually initiating an apoptotic cascade, leading to the formation of mitochondria-associated apoptosis [44].

Impairment of the mitochondrial state initiates appropriate protective mechanisms. For example, the activation of autophagy [45]. Previous studies have shown that autophagy can protect tumor cells from damage caused by external stimuli, a process called protective autophagy. It can also promote damage to tumor cells by external stimuli, a process called destructive autophagy [46, 47]. Therefore, a comprehensive understanding of the role of autophagy may help develop better therapeutic approaches for OS treatment. We suggest that cirsiliol affects the mitochondria to promote the activation of the autophagic system. Furthermore, to demonstrate the effect of autophagy on OS, we added an autophagic agonist and found a more significant increase in apoptosis.

The transcription factor FOXO1 is involved in cell apoptosis, cell cycle and oxidative stress [48]. It was report that FOXO1 can also induce autophagy by upregulating autophagy-related genes, including BECN1, ATG4B, and ATG12 [20], its effects can be regulated by the PI3K/AKT pathway [49]. In normal tumorigenesis, FOXO1 can be driven into the cytoplasm by phosphorylated AKT to undergo degradation [50]. In our study, molecular docking and western blot analysis showed that cirsiliol inhibited the AKT/FOXO1 pathway and inversely regulated the expression of FOXO1, thereby promoting increased autophagy levels.

In summary, our study demonstrated that cirsiliol acts on OS through the AKT/FOXO1 axis by affecting apoptosis and autophagy in OS cells, and works better in U2OS/MTX than in U2OS cells. Thus, our findings suggest that cirsiliol may be a potential clinical candidate for the treatment of both OS and MTX-resistant OS.

Moreover, our study has several limitations, and there is scope for future work. First, the mouse model of osteosarcoma was Cell-Derived tumor Xenograft (CDX) but not Patient-Derived tumor Xenograft (PDX). Therefore, we could not completely simulate a real tumor microenvironment of osteosarcoma in vivo. Second, this study did not explore the altered levels of autophagy in vivo. Finally, the function of cirsiliol in influencing methotrexate resistance of osteosarcoma in vivo needs further investigation.

## Conclusions

In the present study, we revealed the potential anti-cancer features of cirsiliol in OS cells, as evidence from cell proliferation inhibition, G2 arrest, accumulation of autophagosomes, and mitochondrial apoptosis. Meanwhile, in comparison to U2OS, we found that U2OS/MTX was more effective in treatment. Moreover, we propose the mechanism by which cirsiliol reduces the expression of phosphorylated AKT proteins and upregulates FOXO1 to delay OS progression. Thus, cirsiliol is a potential therapeutic candidate for OS, especially for MTX-resistant patients. Future research is expected to improve the prognosis of MTX-resistant patients by combining cirsiliol treatment.

### Supplementary Information

**Additional file 1: Figure S1**. A U2OS cells were treated with different concentrations of cirsiliol, stained with anti-Cyto-C and anti-TOMM20 antibodies and imaged using co-focused microscope imaging. Scale bars:100 µm. B Cyto-C and TOMM20 were quantified using FIJI/ImageJ. Data are expressed as mean ± SD. *p < 0.05; **p < 0.01; ***p < 0.001.

**Additional file 2: Figure S2.** A. Western blot analyses were conducted on lysates extracted from U2OS and U2OS/MTX cells subjected to cirsiliol treatment, employing antibodies against GSK3β, p-S6, S6, and ACTB. B-C. U2OS and U2OS/MTX cells were treated with DMSO, cirsiliol, cirsiliol + SC79, and cirsiliol + siFOXO1. Proliferating DNA expression was assayed. Three independent experiments were performed. Scale bars: 100 µm. Data are expressed as mean ± SD. *p < 0.05; **p < 0.01; ***p < 0.001.

**Additional file 3: Figure S3.** A&C. Apoptosis rates in U2OS cells were measured using flow cytometry and statistically analyzed under treatments DMSO, cirsiliol (20 µM), cirsiliol + SC79, and cirsiliol + siFOXO1. B&D. Immunofluorescence staining was performed to verify the difference in LC3B expression among the DMSO, cirsiliol(20 µM), cirsiliol + SC79, and cirsiliol + siFOXO1 groups. Scale bars: 50 µm. The number of spots was counted on the fluorescent images using FIJI/Image J. Data are expressed as mean ± SD. *p < 0.05; **p < 0.01; ***p < 0.001.

## Data Availability

Please contact author for data requests.

## Abbreviations

OS: Osteosarcoma
CCK-8: Cell counting kit-8
EDU: 5-Ethynyl-2′-deoxyuridine
LC3: Microtubule-associated protein light chain 3
MTX: Methotrexate
Cyto-*C*: Cytochrome *C*
FOXO1: Forkhead Box O transcription factor 1
BCL2: B-cell leukaemia/lymphoma 2
BAX: B-cell leukaemia/lymphoma 2-associated X
BECN1: Beclin1
EBSS: Earle’s balanced salt solution
DMSO: Dimethyl sulfoxide solution
PI: Propidium iodide
IF: Immunofluorescence
IHCP: Immunohistochemistry-paraffin
H&E: Haematoxylin and eosin
BAK: BCL2 antagonist/killer 1
MFN2: Mitofusin 2
ACTB: Beta-actin
MTRC: Mito-Tracker Red CMXRos
LTG: Lyso-Tracker Green
TOMM20: Mitochondrial membrane 20
Cleaved CASP3: Cleaved caspase3
Cleaved CASP9: Cleaved caspase9
PCNA: Proliferating cell nuclear antigen
