# Supplementary figures and images for: Cirsiliol induces autophagy and mitochondrial apoptosis through the AKT/FOXO1 axis and influences methotrexate resistance in osteosarcoma
Source: J Transl Med. 2023 Dec 12;21:907. doi: 10.1186/s12967-023-04682-7 (PMC10714637; doi:10.1186/s12967-023-04682-7)

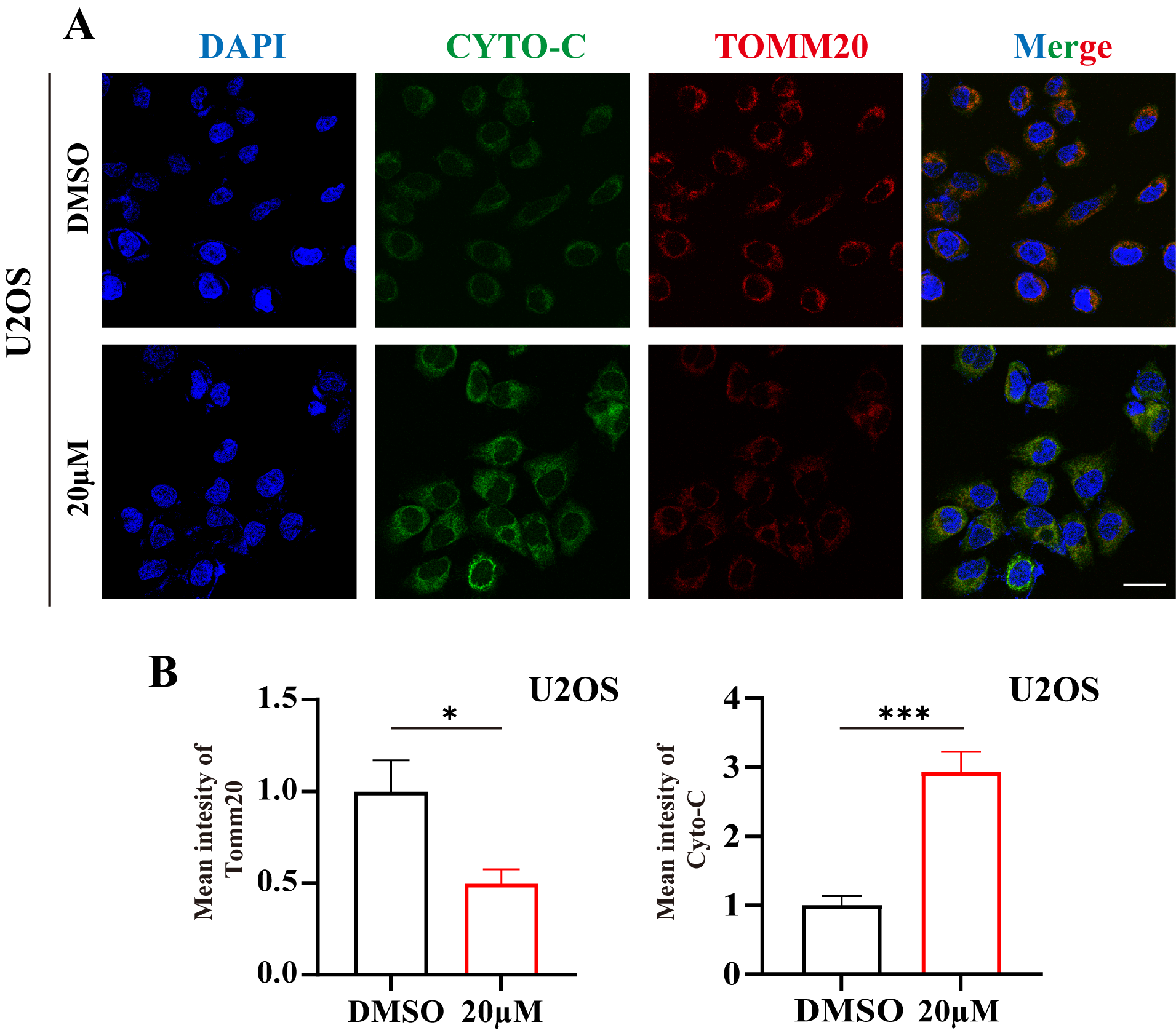

Supplement: Supplementary file 1 — Additional file 1: Figure S1. A U2OS cells were treated with different concentrations of cirsiliol, stained with anti-Cyto-C and anti-TOMM20 antibodies and imaged using co-focused microscope imaging. Scale bars:100 µm. B Cyto-C and TOMM20 were quantified using FIJI/ImageJ. Data are expressed as mean ± SD. *p < 0.05; **p < 0.01; ***p < 0.001. [file 12967_2023_4682_MOESM1_ESM.tif]

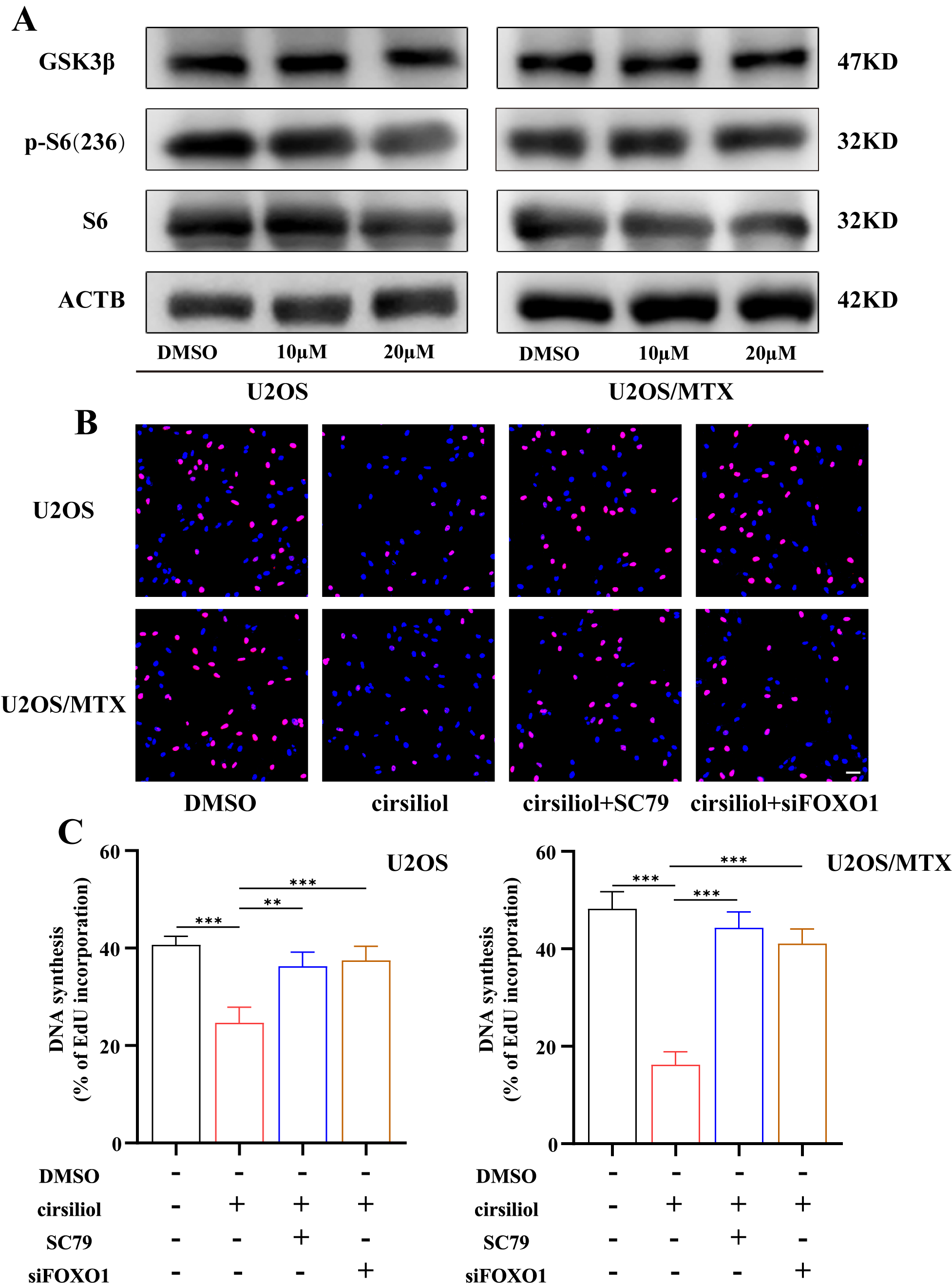

Supplement: Supplementary file 2 — Additional file 2: Figure S2. A. Western blot analyses were conducted on lysates extracted from U2OS and U2OS/MTX cells subjected to cirsiliol treatment, employing antibodies against GSK3β, p-S6, S6, and ACTB. B-C. U2OS and U2OS/MTX cells were treated with DMSO, cirsiliol, cirsiliol + SC79, and cirsiliol + siFOXO1. Proliferating DNA expression was assayed. Three independent experiments were performed. Scale bars: 100 µm. Data are expressed as mean ± SD. *p < 0.05; **p < 0.01; ***p < 0.001. [file 12967_2023_4682_MOESM2_ESM.tif]

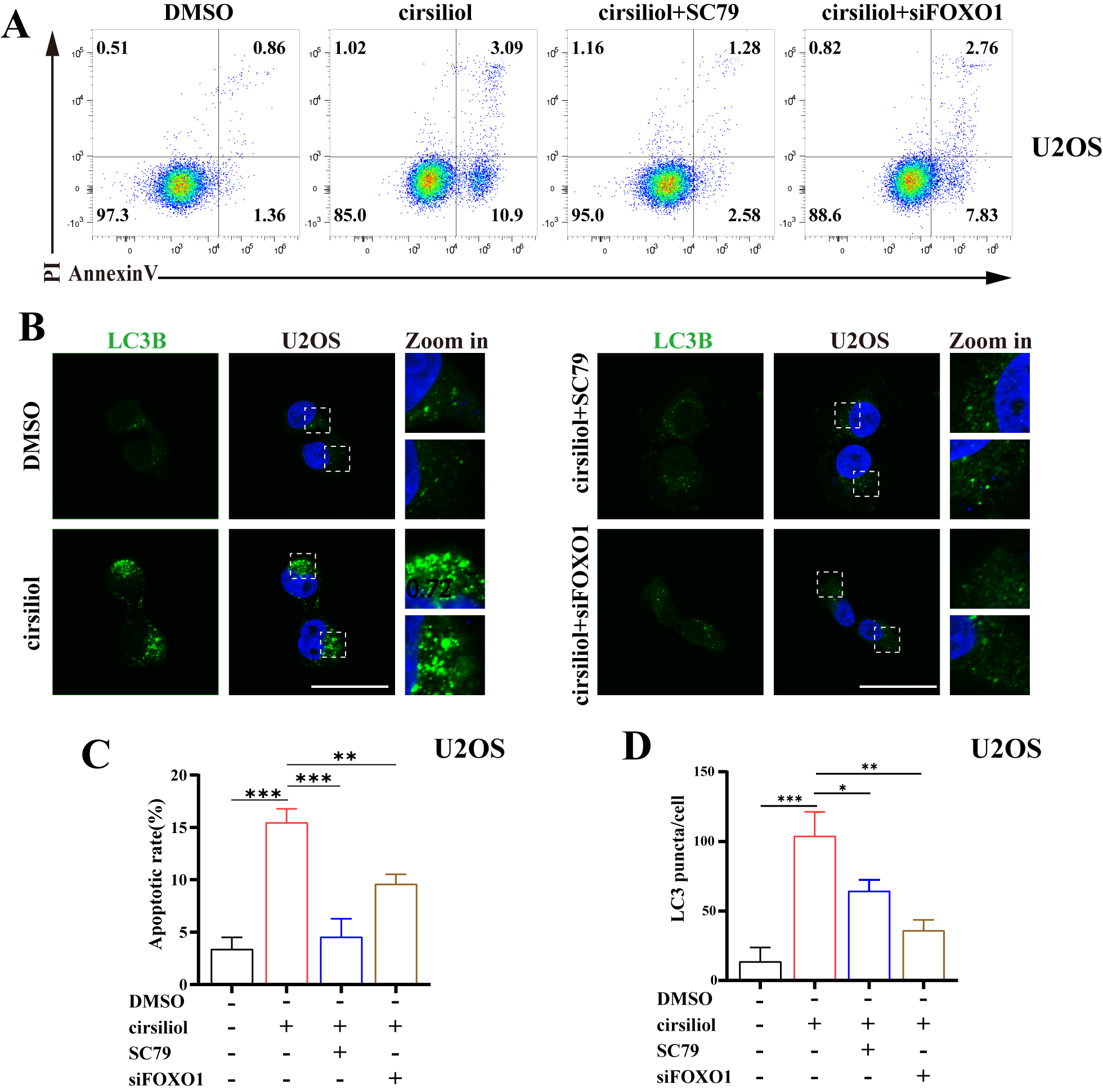

Supplement: Supplementary file 3 — Additional file 3: Figure S3. A&C. Apoptosis rates in U2OS cells were measured using flow cytometry and statistically analyzed under treatments DMSO, cirsiliol (20 µM), cirsiliol + SC79, and cirsiliol + siFOXO1. B&D. Immunofluorescence staining was performed to verify the difference in LC3B expression among the DMSO, cirsiliol(20 µM), cirsiliol + SC79, and cirsiliol + siFOXO1 groups. Scale bars: 50 µm. The number of spots was counted on the fluorescent images using FIJI/Image J. Data are expressed as mean ± SD. *p < 0.05; **p < 0.01; ***p < 0.001. [file 12967_2023_4682_MOESM3_ESM.tif]
